# Questionnaire and tools: clinical powerful instrument in acromegaly diagnosis and management

**DOI:** 10.1007/s40618-022-01782-x

**Published:** 2022-03-23

**Authors:** S. Camerini, A. Wennberg, M. Adriani, B. Martin, R. Vettor, P. Maffei, F. Dassie

**Affiliations:** 1 DIMED, University of Padua, Padua, Italy; 2 Unit of Epidemiology, Institute of Environmental Medicine, Karolinska Institutet, Stockholm, Sweden

**Keywords:** GH, IGF-1, AcroQOL, SAGIT^®^, ACRODAT^®^

## Abstract

**Purpose:**

Acromegaly is a rare chronic disease characterized by systemic comorbidity and reduced quality of life. Although achieving biochemical control has always been the primary goal of acromegaly therapy, recent evidence has shown that the traditional assessment does not adequately capture the complexity of symptoms and patients’ perception. These findings result in the need to improve a fast decision-making process of the clinician, who should not only take into account biochemical–instrumental criteria, but also patients’ symptoms. With the aim of supporting the clinician in the diagnostic and therapeutic decision-making process several disease-specific tools have been developed. The aim of this review is to provide a description of the acromegaly-specific tools, presenting their main features, their application in daily practice, and their efficacy and utility.

**Methods:**

A systematic search of Medline/PubMed, ISI-Web of Knowledge, and Google Scholar databases was done.

**Results:**

Specific instruments and questionnaires have recently been developed to assist clinicians in the assessment of acromegaly. These are either Patient-Reported Outcome tools, such as Acromegaly Quality of Life Questionnaire (AcroQoL) and Pain Assessment Acromegaly Symptom Questionnaire (PASQ), or Clinician-Reported Outcome tools, such as ACROSCORE, SAGIT^®^ and Acromegaly Disease Activity Tool (ACRODAT^®^). Such tools are extremely flexible and, therefore, have been widely adopted by endocrinologists and other professionals, so much so that they have also been included as recommendations in the 2018 international guidelines.

**Conclusion:**

Questionnaires and tools are useful in the management of acromegaly patients. They help clinicians evaluate patients’ symptoms and could assist in the evaluation of disease activity.

## Introduction

Acromegaly is a rare chronic disease characterized by systemic comorbidity and reduced quality of life. Acromegaly can be difficult to detect and is rarely recognized by non-specialist clinicians, resulting in a significant diagnostic delay, which inevitably causes a worsening of the patient's symptoms and general condition [1]. Despite the remarkable improvements recently achieved in acromegaly treatment, patient symptoms and quality of life are typically not completely under control, especially in case of active disease, and even in the case of good biochemical control [2].

Traditionally, the assessment of disease activity has always been based on biochemical (i.e., growth hormone—GH and insulin-like growth factor 1—IGF1) and instrumental (e.g., magnetic resonance imaging—MRI) criteria, but over the years, it has become increasingly clear that these tools have multiple limitations in the diagnosis and ongoing clinical care of acromegaly patients. Although they certainly remain an essential and fundamental part of the clinician's decision-making process, these measures can sometimes be unreliable, difficult to interpret, and may not truly reflect the patient's health status. While several studies have shown an improvement in patient quality of life after treatment, substantial evidence indicates that biochemical control is not necessarily associated with the disappearance of symptoms, leading to the persistence of various impairments (e.g., arthropathy, cardiovascular complications, physical changes) even after disease remission [3, 4]. Optimal care management should therefore go beyond the assessment of biochemical or instrumental data and equally consider patients' perceptions and reported symptoms. In this regard, the patient's personal experience can play an invaluable role. As a result of these findings, specific instruments and questionnaires have recently been developed to assist in the acromegaly patient’s handling process. The goal of this review was to provide a description of the currently available disease-specific instruments for the management of acromegaly by analyzing the studies published in the literature. In particular, it aims to present their main features, their application in daily practice, and their efficacy and clinical utility.

## Methods

We conducted a systematic review of the literature searching the Medline/PubMed, ISI-Web of Knowledge, and Google Scholar databases. The keywords “acromegaly”, “questionnaire”, “tools”, “quality of life”, “QoL”, “ACROQoL”, “SAGIT^®^”, “ACRODAT^®^”, “PASQ”, “SSS”, “ACRO-TSQ”, “ACROSCORE” “Growth hormone”, “GH”, “insulin-like growth factor 1”, “IGF-1”, and “disease activity” were used in various combinations. The search was extended to reference lists of relevant reviews. We excluded duplicate studies. We included prospective, cross-sectional, and basic studies, as well as meta-analyses and review articles, meeting the following criteria: written in English and inherent to the tools and questionnaire in acromegaly topic. Studies were included regardless of their publication status or size. Papers not meeting these criteria were excluded.

## Questionnaires and tools in acromegaly

Specific instruments and questionnaires have recently been developed to support clinical evaluation and management of patients affected by acromegaly. Questionnaire and tools can be classified in Patient-Reported Outcome (PRO) tools, such as Acromegaly Quality of Life Questionnaire (AcroQoL) and the Pain Assessment Acromegaly Symptom Questionnaire **(**PASQ), and Clinician-Reported Outcome (ClinRO) tools, such as ACROSCORE, SAGIT^®^ and Acromegaly Disease Activity Tool (ACRODAT^®^) [3–5]. Such tools are extremely flexible and, therefore, have been widely adopted by endocrinologists and other professionals, so much so that they have also been included as recommendations in the 2018 international guidelines [6]. Some of these tools are also an effective and inexpensive screening method and some of them could be administered to the general population to detect early signs of acromegalic disease. More specifically, they could be used by general practitioners, dentists, or other professionals to identify patients with acromegaly-related signs and symptoms and refer them to an expert for further evaluation [7].

However, tools such as ACRODAT^®^ and SAGIT^®^ find their greatest application in monitoring disease activity during follow-up. Indeed, some of them allow for an extremely thorough examination of the patient, considering both biochemical and instrumental aspects, as well as the patient's perception of symptoms and quality of life. Stated thus, such a comprehensive clinical evaluation could in no way be guaranteed in the traditional outpatient examination due to time constraints [3, 5].

It is also interesting to note that these tools allow standardization of the patient's clinical condition (i.e., an analysis of their health status that goes beyond the clinician's perception). Therefore, one of the main advantages of using instruments such as ACRODAT^®^ and SAGIT^®^ is the possibility of obtaining a complete and standardized assessment of the acromegalic patients, leading to a possible comparison between patients from the same center, but also from different centers [3, 5].

## Patient-reported outcome (PRO) tools

### Acromegaly Quality of Life Questionnaire (AcroQoL)

Patient quality of life (QoL) can be assessed using many different indices. One of the most commonly used is the AcroQoL questionnaire, developed specifically to assess HRQoL (Health-Related QoL) in acromegalic patients by Webb et al. and first validated in 2002 [8].

Originally developed in Spanish and later translated into English, AcroQoL is currently available in 12 languages: Spanish, English, German, Dutch, French, Italian, Greek, Portuguese, Turkish, Swedish, Hungarian, and Polish. Studies using different languages AcroQoL are shown in Table 1. AcroQoL is now considered an excellent tool that clinicians can combine with the outpatient visit to improve patient follow-up [9–55].

**Table 1:** Studies using AcroQOL in different states: year of publication, number of enrolled patients and number of studies

State	Year (number of studies)	Number of patients involved	References
The Netherlands	2007 (1), 2008 (1), 2010 (1), 2011 (1), 2014 (1), 2015 (3), 2020 (2)	402 Collaborative study: 54 + , 27 @, 50§, 80#	[13, 15, 16, 19–21, 31, 32, 34, 40, 43, 46]
UK	2006 (1)–2008 (1)–2017 (1)–2020 (1)–2021 (1)	171 Collaborative study: 58?, 27@	[16, 22, 30, 44, 50]
Italy	2010 (1)–2011 (1)–2018 (1)–2019 (1)–2020 (1)	395	[24, 27, 41, 42, 55]
USA	2008–2015–2018–2020	312 Collaborative study: 106*, 50§	[11, 14, 16, 17, 19]
Brazil	2019 (1)–2020 (1)–2021 (1)	122	[25, 52, 53]
Spain	2017 (1)–2018 (1)–2020	109 Collaborative study: 106*, 58?	[16, 17, 23]
China	2020 (1)–2021 (2)	508	[9, 10, 49]
France	2008 (1)–2020 (1)	122	[12, 45]
Worldwide	2016 (1)–2019 (1)	318	[18, 54]
Germany	2013 (1)–2015 (1)	29 Collaborative study: 80#	[31, 33]
Turkey	2013 (1)–2014 (1)	190	[36, 39]
Swiss	2005 (1)	33	[48]
Mexico	2021 (1)	85	[51]
Taiwan	2019 (1)	272	[26]
Romania	2019 (1)	19	[28]
Poland	2017 (1)	153	[29]
Bulgaria	2015 (1)	212	[57]
Greece	2014 (1)	40	[35]
Venezuela	2014 (1)	28	[37]
Belgium	2007 (1)	291	[49]

*^#^?§@ indicate the same collaborative study

**Fig. 1:**
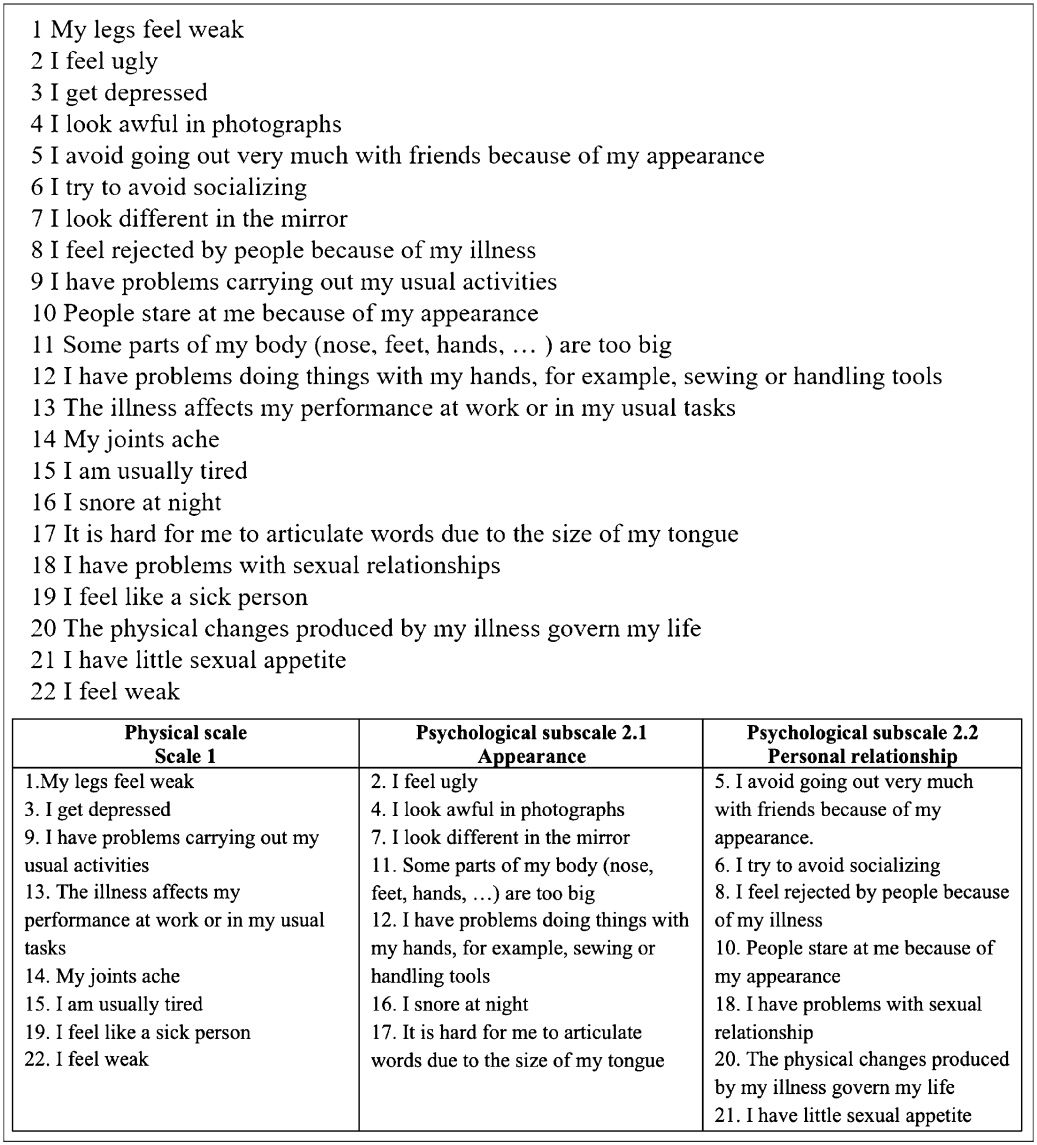
AcroQoL items and subscales

The questionnaire is designed to be easily used in outpatient clinical practice. It is simple, self-reported, and short, taking an average of 7 min to complete. It consists of 22 items, presented in Fig. 1, which the patient must complete by ticking the answer, among those listed, that best describes the frequency of the event or the degree of agreement with the statement expressed by the item [3]. It uses a Likert scale from 0 to 5. The 22 items survey different aspects of the patient's life: 8 items explore the physical aspect, 14 the psychological aspects, 7 items physical appearance, and 7 items are used to determine the personal relationships (Fig. 1). The highest achievable score is 110 (100%) and corresponds to an optimal QoL, while the lowest score is 22 (0%) [3, 8, 16].

In the literature, AcroQoL is one of the most frequently used disease-specific questionnaires, especially when assessing changes in QoL after therapy and/or correlation with normalization of laboratory parameters. As mentioned above, some studies show significant improvement in quality of life in patients assessed before and after initiation of treatment, or higher AcroQoL scores in well-controlled patients compared with those with active disease [3, 4]. Current studies agree on the presence of a decrease in quality of life in acromegaly patients compared to the general population and on the possibility of improvement after therapy. At the same time, other studies also point to the possibility that normalization of quality of life is never achieved, even with optimal biochemical control. In this context, studies such as the one by Paisley et al. from 2007 [22] should be mentioned, but also some more recent ones, such as that of Broersen et al. from 2021 [4], that of Wolters et al. from 2020 [15], or that of Guo et al. from 2021 [9]. All these studies showed the importance of the evaluation of quality of life in acromegaly patients, highlighting numerous factors, such as medical treatments, surgery, radiotherapy, comorbidities, symptoms, and gender as quality of life determinants. Table 2 shows the main QoL determinants in acromegaly patients [9–55]. In particular, in the recent meta-analysis by Broersen et al. [4], the authors found an improvement of HRQoL after acromegaly treatment, but they suggested that the type, frequency, and severity of symptoms and lifestyle factors needed to be considered when evaluating quality of life. The AcroQoL is also often used to validate other questionnaires on well-being and quality of life used in the general population and acromegaly-specific tools, such as the acromegaly treatment satisfaction questionnaire (Acro-TSQ) [14] and SAGIT^®^ [5]. It was also used in comparison with EuroQoL instrument (EQ-5D-5L), Short Form-12 (SF12), Short Form-36 (SF36), EQ-5D-3L and sociodemographic questionnaire to determine a grade of comparison between different tools [5, 9, 14, 16, 17, 20]. Despite the increasing use of the AcroQoL and its recognition as a useful clinical tool, the investigation of the relationship between biochemical disease control and quality of life is still considered a controversial topic, because of the inconclusive consistency of evidence across different studies [56, 57].

**Table 2:** Findings of studies on AcroQoL in acromegaly divided into different topics

Topic	QoL	Findings	References
Treatment of acromegaly	↓ QoL	Patients with active disease vs those with a controlled disease or in remission Active patients at diagnosis During treatment vs after treatment Long term disease controlled (QoL impairment persist even in patients with long-term disease control, duration of biochemical disease control and GH lowering therapy was the predominant factors determining patients QoL) Radiotherapy (QoL worsen progressively in long-term follow-up)	[10, 11, 15, 18, 21, 22, 30, 36–38, 42, 45, 46, 48, 50]
	= QoL	SSA vs PEG (higher body mass index, HbA1c, IGF-1 Z scores are associated with poorer QoL in several domains) LAR with headache	
	↑ QoL	Controlled disease (M > F) in the first year (compared to active patients at diagnosis) Medical and surgery treatment (regardless of diseases activity status) Medical and surgery treatment 3–12 months after therapy no differences between patients with active and controlled disease (no differences between remission/active and discordant results group) Patients treatment satisfactions, treatment efficacy, treatment convenience, treatment of adverse events LAN 120 mg monthly in naïve patients especially on those who have controlled disease LAR treatment (controlled disease > active disease) PEG + SSA vs SSA previously controlled disease PAS LAR treatment and PAS LAR + PEG combination treatment	
Mental health, well-being, illness perception, neurocognitive	↓ QoL	Depression and anxiety (F > M: acromegaly female patients have increased incidence of depression, insomnia and they have reduced social relationship) Antidepressant therapy (acromegaly patients under antidepressant therapy have lower QoL than those not treated) Psychiatrics comorbidities in acromegaly patients compared to patients affected by chronic diseases (acromegaly patients have an increased incidence of anxiety, Mood disturbances (F > M and in those patients with longer disease duration) Poor sleep quality Poorer cognitive function and impaired executive function Negative illness perception even in the long-term remission (negative medication beliefs are related to more negative illness perceptions) Change in body size perception in active disease patients Non-acceptance of the disease Impaired appearance Impaired physical complaint Female sexual dysfunction	[29, 31, 32, 35, 47, 51]
Musculoskeletal	↓ QoL	Musculoskeletal function impairment (muscular function directly correlates with QoL) Vertebral fracture Motor disability Restless leg syndrome (incidence is increased in acromegaly patients) Clinical osteoarthritis of the spine Musculoskeletal pain	[24, 25, 27, 41, 43, 44, 52, 55]
	= QoL	Radiological signs of osteoarthritis of the spine	
	↑ QoL	Muscular rehabilitation	
Dental health	↑ QoL	Satisfactory oral health	[12]
Miscellaneous	↓ QoL	Presence of acromegaly symptoms Presence of acromegaly comorbidities such as diabetes mellitus, cardiovascular comorbidities Female gender vs male gender Older age Active disease	[9, 26, 57]
	↑ QoL	Integration on job market Controlled disease/on remission vs active disease Duration of disease control	

Another relevant application of the questionnaire is to compare the effects of different treatments. Among the most recent studies in this regard is the one conducted by Gu et al. in 2020 [10]. Designed to evaluate the effectiveness of transsphenoidal neurosurgical therapy on patients’ quality of life, this analysis shows the improvement of patients’ quality of life after surgery, and, additionally, that this enhancement is incomplete and independent from the endocrinological remission of the disease. Similarly, Dichtle et al. [11] compared the effects of pegvisomant (PegV) therapy on quality of life compared to somatostatin analogs (SSA) and showed no differences between the two treatments. Beyond the assessment of treatment outcomes, it is also worth mentioning that patients have often expressed their appreciation for the opportunity to discuss their quality of life using this questionnaire, which may help to resolve the issues raised and strengthen the doctor–patient relationship [58]. In fact, a free and open conversation could improve the communication between doctor and patient, since the patient is more inclined to describe his problems, which allows the doctor to adjust the therapeutic process in an appropriate and effective way.

### Pain Assessment Acromegaly Symptom Questionnaire (PASQ)

The PASQ, introduced into clinical practice in 2007, is currently available in 9 languages (English, Italian, Danish, Spanish, Portuguese, Swedish, Dutch, German, and Slovenian) and is the most commonly used questionnaire for the assessment of clinical manifestations in acromegalic patients. In detail, it focuses on some of the most common signs and symptoms: headache, hyperhidrosis, joint pain, asthenia, carpal tunnel syndrome, paresthesia, and swelling of the extremities [59].

The patient is asked to rate the severity of each symptom on a scale from 0 to 8, where 0 is the absence of symptom and 8 is the maximum severity of symptom. Finally, the patient is asked to rate their general health status with a score from 0 to 10, where 0 is optimal health status and 10 is worst health status [59].

In the literature, one of the main applications of the PASQ is the assessment of the patient’s health status after treatment. Notably, the studies by Neggers et al. in 2008 [21] which aimed to shed light on the effects of PegV 40 mg therapy, the study by Caron et al. in 2016 [18], which aimed to rate the effects of lanreotide autogel (LAN), and the study by Broersen et al. in 2021 [4], which showed that both symptoms and HRQoL improve after treatment. The 2015 study by Sievers et al. [60], on the other hand, used the questionnaire to identify the best predictors of response to PegV and showed no association between PRO factors and response to therapy. Lastly, the recent study by Coopmans et al. [13], which investigated the value of biochemical control in monitoring QoL and its changes during pasireotide (PAS-LAR) treatment, shows the absence of a sure and steady correlation between such parameters and an improvement in QoL during PAS-LAR, either as monotherapy or in combination with PegV.

Currently, a shorter version of the PASQ, the Signs and Symptoms Score (SSS), is often used in clinical practice. This is a pathology-specific questionnaire consisting of 5 items, each of which relates to a symptom commonly associated with acromegaly: headache, hyperhidrosis, joint pain, asthenia, and soft tissue swelling. As with the PASQ, the patient is asked to indicate the severity of the symptom with a score from 0 to 8. The maximum score that can be achieved is 40 and corresponds to an extremely severe disease state [59].

### Acromegaly Treatment Satisfaction Questionnaire (ACRO-TSQ)

Current evidence shows that patient quality of life depends not only on the effects of acromegaly, but also on the effects of the therapy they receive. In this regard, the Acro-TSQ, a PRO questionnaire specifically designed for use in regular follow-up of patients treated with injectable SSA, was developed. This 2019-validated instrument [61] aims to assess the acromegalic patient, taking into account not only the symptomatology associated with the disease, but also the patient's self-perception of the current therapy (adverse effects, overall satisfaction, usefulness, convenience of use). Items from the questionnaire explore the following parameters: symptoms, gastrointestinal interference, treatment satisfaction, injection site interference, emotional reaction, and treatment convenience [14].

Although the questionnaire has only recently been introduced, it has already been widely used in the literature to assess the impact of therapy with SSA on the patient. In this context, the 2021 study by Fleseriu et al. is particularly worth mentioning. It analyzed the side effects of treatment with octreotide LAR (OCT LAR—stable dose for more than 6 months) or LAN LAR (stable dose for more than 4 months) in patients with biochemically well-controlled disease. Data from this study showed how most subjects (approximately 75%) experienced both treatment side effects and acromegaly-related symptoms during therapy, and that these side effects severely affected their daily lives, including work activities and leisure time [62]. Similar in aim and evidence is the 2020 study by Geer et al. [63], which found that even while receiving a steady regimen of first generation injectable somatostatin analogues, most patients report an incomplete control of symptoms that disrupt daily life, leisure and work. The Acro-TSQ was also used to observe whether there was agreement between patients’ and clinicians’ perceptions. More specifically, the study weighed the level of clinician perceptions regarding the frequency and severity of patients’ symptoms and their improvement after treatment [64].

### Enlargement of the Extremities Questionnaire

The 2012 work by Rosaro and Casolari [65] proposes a short screening questionnaire with the aim of early detection of acral growth. The questionnaire contains only two questions: (1) Have you noticed an increase in your size over the last 5 years? and (2) In the last 5 years, have you had to have your rings adjusted because they became too tight? Underlying the study there is the awareness that many of classical acromegaly signs and symptoms are actually very common, instead acral growth in adults is an extremely rare event and, when present, represents one of the main reasons for the patient to consult a physician. Therefore, searching for acral growth in the general population and performing specialist examinations in patients who test positive would lead to earlier acromegaly diagnosis [65].

### Acromegaly Comorbidities and Complaints Questionnaire (ACCQ)

ACCQ is an 8-item questionnaire that evaluates acromegaly signs, symptoms, and comorbidity frequency and intensity. Each item is answered on a 0–3 scale measuring the severity of symptoms (absent, mild, moderate, severe). Results range between 0 and 24, and, based on this result, patients are classified into categories: very mild constraint (≤ 8), mild-to-moderate impact (9–16), severe impact (≥ 17). Psaras et al. in a validation study evaluated signs, symptoms, and comorbidities after treatment and their impact on acromegaly patients’ quality of life. The authors determined that joint impairment influences quality of life among men, while women perceived late effects of hypertension as a manifest health threat [66].

### Acromegaly Comorbidities Questionnaire (Acro-CQ)

Acro-CQ is a 22-item questionnaire specifically developed for patients affected by acromegaly to assess comorbidities (metabolic disorders, cardiovascular disease, neoplastic disorders, intestinal diverticulosis/diverticulitis, gallbladder and kidney stones, goiter, carpal tunnel syndrome), ongoing treatment and family history of pituitary adenoma. The study by Guaraldi et al. demonstrates that the questionnaire is inexpensive, clear, comprehensive and easy to apply in clinical practice, but also identifies the long time required to complete the questionnaire itself as its major limitation, especially in those patients with multiple comorbidities [67].

## Clinician-reported outcome (clinro) tools

The CLINRO tools main characteristics and their applications are shown in Table 3.

**Table 3:** Characteristics of the main clinician-reported outcome tools

Tool	Characteristics	Applications	References
ACRODAT	-Online tool; -5 parameters selected: IGF-1 levels, comorbidities, tumor status, symptoms, HRQoL; -3 levels of disease: stable (S), moderate (M- DA), and significant (S- DA)	Follow-up	[3]
SAGIT	-5 parameters selected: signs/symptoms (0–4 pt); comorbidities (0–6 pt); GH nadir/random (0–4 pt); IGF-1/ULN (0–3 pt); tumor size (0–5 pt) -Higher score = worst disease	Follow-up	[5, 69]
ACROSCORE	-Presence of 6 early symptoms: diabetes mellitus type 2 (1 pt); hyperhidrosis (2 pt); thyroid hyperplasia (3 pt); carpal tunnel syndrome (1 pt); dental diastasis (4 pt); colon polyps (3 pt) -Final score = risk of being affected by acromegaly (0 = low risk, 1–5 = moderate risk, > 5 = high risk)	Early diagnosis (identify common manifestations of acromegaly)	[7]

### Acromegaly disease activity tool (ACRODAT^®^)

ACRODAT^®^, validated in 2017, is a tool to assist clinicians in determining disease activity in patients with acromegaly. The main advantage of this tool is that it allows a complete evaluation of the pathology, considering not only the clinical aspects of the disease, but also biochemical aspects and tumor mass characteristics simultaneously [3].

ACRODAT^®^ was developed by a panel of acromegaly experts (endocrinologists and neurosurgeons). Ten experts defined parameters to monitor disease activity focusing on clinical practice and health status, and, for each parameter, they determined three levels of severity. Based on expert parameters different hypothetical clinical scenarios were assessed and judged from other acromegaly experts to validate the 5 key parameters. Specifically, ACRODAT^®^ uses the 5 key parameters (IGF-1 levels, comorbidities presence, tumor status, symptoms and health-related quality of life) that have been identified as the best predictors of disease activity and are divided into categories that indicate severity. Using these parameters, ACRODAT^®^ defines three levels of disease: stable (S), moderate (M-DA), and significant (S-DA). These stages are then defined by colors: green for S, orange for M-DA, and red for S-DA. The five parameters selected were: IGF-1 levels (red: IGF-1 ULN > 1.2); tumor status at current MRI (red: significant increase in tumor size and/or invasiveness is observed since prior MRI and/or impaired vision); presence of GH excess comorbidities (i.e., diabetes mellitus, sleep apnea, and cardiovascular disease); symptoms (red: SSS > 4 or more symptoms rated major than 6); and health-related QoL impairment as AcroQoL results (red: AcroQoL < 40) [3].

The validity of the tool seems to be confirmed not only by endocrinologists, but also by patients. More specifically, the 2019 study by Jackson et al. aimed to determine the definition of “active disease” from the patients’ perspective and to measure the importance of the various parameters included in ACRODAT^®^. The resulting data show how all 5 parameters in the tool were considered influential in assessing disease activity from the patient's perspective and how quality of life and “patient-centered” parameters played a dominant role [68]. For ease of use, ACRODAT^®^ is available as on-line tool. This type of quick screening tool is useful but also has major limitations that must be considered, such as the fact that other factors, besides those considered, could also play an important role. It is therefore clear that specialists should use ACRODAT^®^ as a support for their own clinical knowledge and not as a stand-alone tool [3]. ACRODAT^®^ development was financially supported by Pfizer.

### SAGIT^®^

The acronym SAGIT^®^ (S Signs and symptoms, A Associated comorbidities, G GH concentration either assessed as GH nadir after oral glucose tolerance test or GH random or series, I IGF-1 levels and T Tumor) refers to a pathology-specific tool first introduced in 2015 [5, 69] that aims to provide a tool to assist clinicians in assessing the acromegalic patient after initial diagnosis and during follow-up. For every parameter, this tool considers the following characteristics:

- Signs and symptoms: headache, sweating, joint symptoms and acral overgrowth; score ranges from 0 to 4, according to number of symptoms endorsed;
- Associated comorbidities: impaired glucose metabolism, hypertension, sleep apnea, heart disease, hypopituitarism, active malignancies; score ranges from 0 to 6 according to number of comorbidities endorsed;
- GH: 5 ranges of concentration of GH nadir (< 0.4, 0.4–1.0, 1.0–2.5, 2.5–5, > 5 mcg/L) or random GH (< 1, 1–2.5, 2.5–5.0, 5.0–10, > 10 mcg/L); score ranges from 0 to 4 according to different ranges;
- IGF-1: 4 ranges of concentration; score ranges from 0 to 3 based on different IGF-1 ULN (normal, < 1.3, 1.3–2, ≥ 2);
- Tumor: 6 categories of tumor size (pituitary mass not visible, microadenoma intrasellar, macroadenoma intrasellar, extrasellar adenoma < 40 mm, invasive adenoma, giant adenoma ≥ 40 mm); score ranges from 0 to 5 [5, 69].

In 2019, through an interim analysis study of the data used in the validation phase of SAGIT^®^, Giustina et al. showed how there is a significant discrepancy between disease status in the clinician’s opinion, actual disease activity, hormonal control, and therapeutic decisions. In this regard, SAGIT^®^ could be useful as an aid in the clinician’s decision-making process by guiding them in assessing the level of disease activity and consequently suggesting whether or not a change in current treatment is necessary [5].

Recently, international SAGIT^®^ validation study was published. In this study patients were divided into active or controlled one based on CGE-DC (clinical global evaluation of disease control) questionnaire, clinicians’ therapeutic decisions and guidelines recommendations. Results revealed that at baseline the components S, G, I and T were statistically different in patients with active disease compared with those with control one, the same analysis at the end of the study (after 2 years) showed that only components G and I differed between the two groups. A following classification and regression tree analyses (CART) showed that components I and G for controlled acromegaly at CGE-DC and all the SAGIT® components for clinicians’ therapeutic decisions were those that define acromegaly disease status. Finally, patients classified on active and controlled ones based on acromegaly guidelines differed only for the component [70].

As mentioned in the description of ACRODAT^®^, one of the main advantages of the tool is the fact that it allows a comprehensive disease investigation considering clinical aspects, biochemical values, and tumor characteristics.

The tool has been welcomed by experts. As shown in the 2014 study by Giustina et al., most endocrinologists considered SAGIT^®^ useful, both for research purposes and for the decision-making in clinical care, including treatment process, describing it as concise, simple, unbiased, and informative [5, 69, 70].

The questionnaire, however, does not take into account the patient's perception of quality of life (as ACRODAT^®^ does). In this sense, using the SAGIT^®^ in conjunction with a PRO questionnaire, such as AcroQoL, is recommended [5]. Notably, clinicians should be aware that SAGIT^®^ cannot be used in the evaluation of patients under pegvisomant treatment because GH levels may be altered in these patients [5, 69, 70]. SAGIT^®^ development was financially supported by Ipsen.

### Acroscore

Unlike the tools outlined in previous paragraphs, the ACROSCORE questionnaire, published in 2016, is not designed for the follow-up of patients, but instead aims to identify common early manifestations of acromegaly, and thus assist the non-specialist clinician in raising diagnostic suspicion of disease [7].

It is essential to state that the score does not account for some of the most characteristic signs of the disease, such as acral overgrowth and other phenotypic changes. Indeed, these signs generally occur at fairly advanced stages, whereas the purpose of the ACROSCORE is to identify patients with acromegaly at the earliest possible stage. Therefore, only signs, symptoms, and comorbidities with an early onset are included into the score [7].

The selection of the criteria considered by the tool was based on a multicentric study of comparison between the clinical characteristics of patients with acromegaly and healthy subjects. Multivariate logistic models were then used to calculate the value of each symptom, so that a corresponding score could be assigned to each symptom: diabetes mellitus type 2 = 1 point, hyperhidrosis = 2 points, thyroid hyperplasia = 3 points, carpal tunnel syndrome = 1 point, dental diastasis = 4 points, and colon polyps = 3 points. The sum of the points results in the final score (maximum 14), which allows the patient to be classified into different categories according to the risk of being affected by acromegaly: low risk (0), moderate risk (1–5), high risk (> 5). In summary, the goal of ACROSCORE is to detect the disease at an early stage, which not only allows for the possibility to improve the patient's therapeutic management and outcome, but could also lead to a positive economic impact by reducing the occurrence of comorbidities associated with prolonged and poorly controlled disease [7].

## Conclusion

The use of the instruments analyzed in this review can pursue different objectives, depending on the type of instruments used, but in any case they prove to be efficient and successful.

As mentioned above, acromegaly is a complex disease characterized by signs, symptoms and multi-organ comorbidities that lead to an inevitable impairment of the patient's quality of life. These impairments become more severe as disease activity increases, leading to deterioration in the patient’s clinical condition, increased risk of mortality and higher treatment costs to the nationals health systems.

Now that the health of acromegaly patients has been shown to improve with appropriate treatment, it is clear that early onset therapy is required, which must also be effective and as individualized as possible.

To achieve this goal, as well as early diagnosis, it is critical to ensure an integrated assessment of the patient, which should include signs and symptoms, biochemical parameters, magnetic resonance, quality of life, and patient perception.

These findings lead to the need to enrich the physician's decision-making process, which, however, may require a large amount of time during the outpatient visit and a great deal of effort for the physician, who must consider a large number of variables, leading to increased workload and difficulty in patient care.

In reviewing the studies currently available in the literature, it was found that the use of such instruments can be extremely useful in daily clinical practice, as they facilitate the physician’s assessment of the patient and improve the diagnosis rate.

In conclusion, both patients’ Reported Outcome tools and Clinician-Reported Outcome tools are useful in daily clinical practice and can be used by spokes and hub hospital personal to standardized evaluation of quality of life and patients' perspective.

## Data Availability

Not applicable.
